# The influence of snoring, mouth breathing and apnoea on facial morphology in late childhood: a three-dimensional study

**DOI:** 10.1136/bmjopen-2015-009027

**Published:** 2015-09-08

**Authors:** Ala Al Ali, Stephen Richmond, Hashmat Popat, Rebecca Playle, Timothy Pickles, Alexei I Zhurov, David Marshall, Paul L Rosin, John Henderson, Karen Bonuck

**Affiliations:** 1 Applied Clinical Research & Public Health, Dental School, Wales, UK; 2 School of Computer Science & Informatics, Cardiff University, Wales, UK; 3 Avon Longitudinal Study of Parents and Children, University of Bristol, Bristol, UK; 4 Department of Family and Social Medicine, Albert Einstein College of Medicine, Bronx, New York, USA

**Keywords:** ALSPAC, sleep disordered breathing, snoring, apnoea, mouth-breathing, three-dimensional imaging

## Abstract

**Objective:**

To explore the relationship between the prevalence of sleep disordered breathing (SDB) and face shape morphology in a large cohort of 15-year-old children.

**Design:**

Observational longitudinal cohort study

**Setting:**

Avon Longitudinal Study of Parents and Children (ALSPAC), South West of England.

**Participants:**

Three-dimensional surface laser scans were taken for 4784 white British children from the ALSPAC during a follow-up clinic. A total of 1724 children with sleep disordered breathing (SDB) and 1862 healthy children were identified via parents’ report of sleep disordered symptoms for their children. We excluded from the original cohort all children identified as having congenital abnormalities, diagnoses associated with poor growth and children with adenoidectomy and/or tonsillectomy.

**Main outcome measures:**

Parents in the ALSPAC reported sleep disordered symptoms (snoring, mouth breathing and apnoea) for their children at 6, 18, 30, 42, 57, 69 and 81 months. Average facial shells were created for children with and without SDB in order to explore surface differences.

**Results:**

Differences in facial measurements were found between the children with and without SDB throughout early childhood. The mean differences included an increase in face height in SDB children of 0.3 mm (95% CI −0.52 to −0.05); a decrease in mandibular prominence of 0.9° (95% CI −1.30 to −0.42) in SDB children; and a decrease in nose prominence and width of 0.12 mm (95% CI 0.00 to 0.24) and 0.72 mm (95% CI −0.10 to −0.25), respectively, in SDB children. The odds of children exhibiting symptoms of SDB increased significantly with respect to increased face height and mandible angle, but reduced with increased nose width and prominence.

**Conclusions:**

The combination of a long face, reduced nose prominence and width, and a retrognathic mandible may be diagnostic facial features of SBD that may warrant a referral to specialists for the evaluation of other clinical symptoms of SDB.

**Strengths and limitations of this study**

- The strengths of this study are its large sample size based on a population cohort of UK children that is broadly representative of the general population. In addition, the children were of the same age (15 years) making comparisons stronger. Furthermore, the possible confounding effect of obesity was ascertained. Finally, the imaging method used in the study is valid and therefore the methods are transferrable to other population groups.
- A limitation of this study is that sleep disordered breathing (SDB) data are based on parent-report of SDB symptoms rather than a polysomnogram (PSG), which is considered the ‘gold standard’ for assessing SDB. However, the time, expense, possible selection bias of those undergoing PSG and possible methodological changes over time rendered it unfeasible for epidemiological purposes in a large longitudinal cohort study. However, the five patterns of symptoms of SDB defined in this study are correlated with the outcomes of polysomnogram examinations and were found to be reliable.

## Introduction

Sleep-disordered breathing (SDB), including obstructive sleep apnoea (OSA), is highly prevalent in the general population. The most common symptoms are primary snoring, mouth breathing and repetitive periods of cessation in breathing during sleep, termed apnoeas or reductions in the amplitude of a breath, known as hypopneas.^1^ American Academy of Otolaryngology-Head and Neck Surgery (AAO-HNS) defines SDB as “an abnormal respiratory pattern during sleep and includes snoring, mouth breathing, and pauses in breathing”.^2^

SDB is a subtle disorder of early childhood, and may have serious consequences for long-term health, especially among children with macroglossia and retrognathia.^3^ ^4^ There have been few investigations concerning the prevalence of SDB in children. Snoring has been reported in 10% of preschool children.^5–7^ The prevalence of parent-reported snoring is estimated to be 7.5% for 2–18-year-olds,^8^ while the prevalence of mouth breathing in young children ranges from 3% to more than 50%,^9–11^ and the prevalence of OSA is reported to range from 0.7% to 4% among 2–18-year-olds.^12^ ^13^

The prevalence of SDB symptoms is reported by Bonuck *et al*,^14^ who conducted the first study on the natural history of snoring, mouth breathing and apnoea in a population-based cohort across a key 6-year period, in the development of SDB symptoms. The prevalence of ‘Always’ snoring range=3.6%–7.7%, ‘habitually’ snoring range=9.6%–-21.2%, the prevalence of apnoea (‘Always’) was 1–2% and ‘Always’ mouth breathing ranged from 2.1% to 7.6%.^14^

The current view is that adenotonsillar hypertrophy is the major cause of SDB in otherwise healthy children.^15–17^ Adenotonsillar hypertrophy is associated with nasal obstruction, resulting in breathing problems and leading to disturbed patterns of sleeping, eating, swallowing and speaking.^18^ Consequently, the primary therapy for children with SDB is adenotonsillectomy.^19^

It is possible that obesity is a risk factor of SDB. Rudnick *et al*^20^ reported that children with SDB who undergo adenotonsillectomy are more likely to be obese than children seen in a general paediatric clinic, and that African-American children who are obese are more likely to have SDB. Verhulst *et al*^21^ reviewed the literature on the prevalence, anatomical correlates and treatment of SDB in obese children. They concluded that obese children are at a higher risk of developing SDB, and suggest that in such children, adiposity and upper airway factors, such as adenotonsillar hypertrophy, both moderated the severity of SDB. In a direct response to this review, Kohler and van den Heuvel^22^ argue “We believe, however, that the available studies do not support a straightforward association of overweight or obesity with increased prevalence of SDB. Rather, the available data are clearly equivocal mainly due to methodological differences between the previous studies”. Kohler and van den Heuvel examined other factors that may moderate the relationship between overweight or obesity, and prevalence of SDB in children, particularly ethnicity and age. In a more recent case–controlled study, Tripuraneni *et al*^23^ conclude that the degree of obesity does not linearly predict the severity of OSA in children; however, obese children may have worse symptoms of OSA (diagnosed by polysomnography (PSG)) than normal-weight children.

The concept that nasal obstruction and associated mouth breathing affects craniofacial development and morphology continues to be controversial.^24^ A number of craniofacial anomalies including maxillary and mandibular retrognathia, enlarged tongue, soft palate, adenotonsillar hypertrophy and an inferiorly positioned hyoid bone, may be associated with decreased posterior airway space and restriction of the upper airway, promoting apnoeas and hypopnoeas during sleep.^25^ Cessation of airflow may develop during OSA because of anatomic obstruction of the upper airway related to obesity, and excessive tissue bulk in the pharynx.^1^

Our previous studies on ALSPAC children with breathing disorders (atopy, allergic rhinitis and asthma) show that the previously mentioned breathing disorders may have influenced face shape to varying degrees.^26^ ^27^ Atopic and allergic children had an increased total face height on an average of 0.6 mm when compared to controls, whereas asthmatic children had a shorter mid-face height (0.4 mm) compared to a control group drawn from the same population.

Linder-Aronson^28^ evaluated children who had adenoid hyperplasia and concluded that nasal obstruction may alter facial growth, for which the term ‘long or adenoidal face’ was coined. He found that children with large adenoids usually have longer and narrow faces, lower tongue position, anterior open bite, narrow upper jaw and steep mandibles with a more backward position. Linder-Aronson *et al*^29^ hypothesised that adenotonsillar hypertrophy in children induces mouth breathing, disrupting the balance of labial, lingual and cheek muscles, resulting in facial anomalies. Tomer and Harvold,^30^ and Vickers,^31^ suggest that nasal obstruction causes changes in muscular function, conditioning dentofacial anomalies. Children with enlarged tonsils may have a larger anterior total and lower facial height, a more retrognathic mandible, proclined upper incisors, retroclined lower incisors and a large overjet.^32^ Other facial anomalies that may potentially be associated with SDB include increased anterior face height, incompetent lip posture, increased mandibular plane angle and V-shaped maxillary arch.^33^ Nasal obstruction associated with mouth breathing may lead to a downward and backward rotation of the mandible associated with increased anterior face height.^24^ A recent systematic review and meta-analysis comparing healthy controls versus children with OSA and primary snoring, matched for gender,^34^ provides limited statistical support for an association between OSA and cephalometric measurements in children aged 0–18 years. The meta-analysis used data extracted from randomised controlled trials, case–controls and cohort studies, with relatively small aggregated samples. The maximum sample sizes were n=87 cases with OSA and n=113 healthy controls. Relative to the controls, children with OSA and primary snoring were found to exhibit (1) a significantly increased mandibular plane angle to the cranial base (ANB angle); and (2) a significantly reduced upper airway sagittal width. An increased ANB angle of less than 2^o^ was, however, regarded as having marginal clinical significance. The study concludes that “larger well controlled trials are required to address the relationship of craniofacial morphology to paediatric sleep-disordered breathing”, providing a direction and rationale for the current study.

## Purpose

The purpose of this study is to determine the extent to which SDB (symptomised by snoring, mouth breathing or OSA) is statistically related to face shape measurements among children in late childhood (15 years of age). Based on the literature, we hypothesise that the following face shape measures might be predictors of the incidence and/or patterns of severity of SDB: (1) increased face height; (2) smaller nose prominence; (3) smaller mandibular prominence; and (4) smaller maxillary prominence.

## Methods

### Subjects and outcomes

The children who participated in this study were Caucasian representatives of the UK population. They were recruited from the Avon Longitudinal Study of Parents and Children (ALSPAC), which is designed to explore how the individual's genotype is influenced by environmental factors impacting on health, behaviour and development of children.^35^ The initial ALSPAC sample consisted of 14 541 pregnancies with an estimated date of delivery between April 1991 and December 1992. Oof the initial 14 541 pregnancies, all but 69 had known birth outcome. Of these 14 472 pregnancies, 195 were twins, 3 were triplets and 1 was a quadruplet pregnancy; meaning that there were 14 676 fetuses in the initial ALSPAC sample. Of these 14 676 fetuses, 14 062 were live births and 13 988 were alive at 1 year.

SDB was assessed through parental reports of SDB's hallmark symptoms (snoring, apnoea and mouth breathing) when each child was 6, 18, 30, 42, 57, 69 and 81 months of age. Since objective sleep evaluation and polysomnography data were not available, the assessment of the incidence of SDB was not fully diagnostic. Based on Freeman and Bonuck^36^ cluster analysis of SDB symptoms, five categories of SDB, based on the severity of symptoms reported by the parents over time, were classified as follows:

- Asymptomatic (healthy);
- Early snoring, with peak symptoms at 6 months;
- Early snoring, with peak symptoms at 18 months;
- Late snoring and mouth breathing, but remained asymptomatic until 4 years old; and
- Severe and sustained symptoms of SDB throughout childhood.

During 2006 and 2007, the cohort was re-called when the children were 15 years of age. We excluded from the original cohort all children identified as having (1) congenital abnormalities; (2) diagnoses associated with poor growth; and (3) children with adenoidectomy and/or tonsillectomy, because we focused on cases of SDB not secondary to congenital or other medical complications. The total sample size used in this study was 3586 (1693 males and 1893 females).

**Figure 1:**
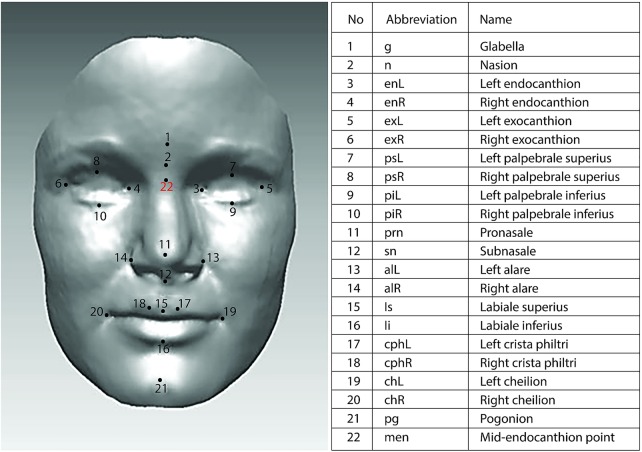
Facial soft tissue landmarks.

The laser scanning system consisted of two high-resolution cameras (Minolta VIVID 900 Optical Digitizers) operating as a stereopair. The system can acquire 307 200 (640×480) data points as the x-coordinates, y-coordinates and z-coordinates of the surface scanned, with an average reported manufacturing accuracy of 0.1 mm (±0.2 mm).^37^ A strict protocol for capturing facial soft tissue morphology was applied in this study. Children sat on an adjustable stool and were asked to look at a Bristol red glass heart hung from the ceiling to simulate natural head posture (NHP). NHP was adopted because it has been shown to be clinically reproducible.^38–40^ Children were also instructed to swallow hard and to keep their jaws relaxed just before the scans were taken. If a patient moved between scans, the procedure was repeated. The scanning took approximately 8 s per child. A locally developed algorithm implemented as a macro in Rapidform software (INUS Technology Inc, Seoul, South Korea) was used to process, register and merge the left and right facial scans of each individual.^41^ Prior to merging, the scanning accuracy was checked. At least 90% matching of the overlap area of facial halves, with an error ≤0.75 mm, was deemed to be clinically acceptable. Facial images were normalised to natural head posture with the origin set at mid-endocanthion point, because this is the most stable point with respect to the growth of the face.^42^ The 21 soft tissue landmarks shown in figure 1 were manually identified on each facial shell using the Rapidform software.^43^ The precision of measuring the landmarks was <1.0 mm for both intraexaminer and interexaminer assessments. The 17 face shape variables calculated from the landmarks are listed in table 1.

**Table 1:** Variables operationalised from facial landmarks (R=Right; L=Left)

Variables	Landmarks	Units
Outer eyes distance	exR-exL	mm
Inner eyes distance	enL-enR	mm
Lower face height	Is-pg	mm
Mandible angle	g-men-pg	Degrees
Maxilla angle	n-sn-pg	Degrees
Mid-face height angle	exR-pg-exL	Degrees
Mid-face height	Is-men	mm
Mid-face height	sn-men	mm
Mid-face height	n-sn	mm
Nose prominence angle	n-prn-sn	Degrees
Nose prominence	prn-sn	mm
Nose width	alL-alR	mm
Philtrum angle	prn-sn-Is	Degrees
Total face height	pg-n	mm
Total face height	pg-g	mm
Total face height	li-men	mm
Total face height	pg-men	mm

Surface-based average faces were constructed separately for those with SDB (1724) and healthy children (1862). In this study, facial averaging was implemented using a template face (randomly chosen from the sample) and by calculating point-wise mean coordinates in the direction nearly perpendicular to all faces.^41^ The resulting average face was then used as a new template and the averaging was repeated. Three iterations were performed in order to get average faces with good accuracy for children with SDB and healthy children.^41^

The average face of children with SDB was compared with the average face of healthy children, by superimposing them on the mid-endocanthion point using a best-fit registration.^41^ ^44^ Within each of the average faces there is a variability in the anteroposterior, vertical and transverse dimensions. For this reason, positive and negative changes are produced between the different study groups. Colour maps were used to illustrate this with a tolerance level of 0.1 mm to highlight significant topographical facial differences.

### Statistical analysis

The statistical analysis was conducted using IBM SPSS V.20. The steps undertaken were: a χ^2^ test was used to examine the association of SDB status with gender. Second, an independent sample t test was used to compare body mass index (BMI) in SDB status and analysis of variance (ANOVA) was then used for SDB groups. Third, factor analysis was used to reduce the 17 facial angles and measurements into a smaller number of facial dimensions. Logistic regression was then used to examine the relationship between the facial dimensions and SDB status. Finally, SDB groups were plotted in box plots and tested for significance using one way ANOVA.

## Results

### Demographic summary

The total sample consisted of 3586 children at age 15, of whom 52.8% were female. The demographic summaries of the 1724 children with SDB symptoms and 1862 asymptomatic children are summarised in table 2. The five levels of SDB severity, ranging from asymptomatic to the persistent group, were represented by 52.8%, 15.6%, 9.9%, 16.2% and 5.3% of the total sample, respectively.

**Table 2:** Demographic summary of the sample

	Percentage (n) or Mean (SD)
Gender (% (n) Female)	52.8 (1893/3586)
BMI (kg/m²)	21.4 (3.5)
SDB status	
Asymptomatic	52.8 (1862)
Early snoring, peak symptoms at 6 months	15.6 (599)
Early snoring, peak symptoms at 18 months	9.9 (354)
Late snoring and mouth breathing	16.2 (580)
Severe and sustained symptoms of SDB	5.3 (191)

BMI, body mass index; SDB, sleep disordered breathing.

SDB was not significantly associated with gender (χ^2^ p=0.282). The proportion of boys and girls with SDB was 49% and 47.2%, respectively. BMI was associated with SDB (mean difference (95% CI) in BMI 0.28 (−0.51 to −0.06), p=0.012). The SDB children's BMI (21.44) was higher than that in healthy children (21.15). However, BMI did not differ within SDB groups (ANOVA p=0.100).

### Comparison of face shape dimensions

The descriptive statistics (means, SDs and 95% CI) for the 17 face shape variables are presented in table 3. Significant mean differences in facial measurements in those with SDB were: an increased lower face height (Is-pg); a decrease in nose prominence (prn-sn); a decrease in nose width (alL-alR) and an increase in mandible angle (g-men-pg), indicating a retrognathic mandible in those with SBD.

**Table 3:** Descriptive statistics for face shapes

		Non-SDB		SDB			
	Dimension	M	SD	M	SD	ΔM	CI
1	Total face height (pg-men)	93.50	5.63	93.75	5.56	0.25	(−0.61 to 0.11)
	Total face height (pg-g)	113.46	6.12	113.80	6.10	0.33	(−0.73 to 0.06)
	Total face height (pg-n)	101.46	6.17	101.85	6.22	0.39	(−0.80 to 0.01)
	Lower face height (Is-pg)	36.54	3.52	36.83	3.60	0.28	(−0.52 to −0.05)
	Total face height (li-men)	74.73	4.69	74.97	4.74	0.24	(−0.55 to 0.06)
	Mid-face height (Is-men)	61.69	4.00	61.66	3.96	0.03	(−0.23 to 0.29)
	Mid-face angle (exR-pg-exL)	49.67	2.60	49.41	2.68	0.26	(−0.08 to 0.43)
2	Outer eyes distance (exR-exL)	87.68	3.91	87.33	4.11	0.34	(−0.08 to 0.60)
	Inner eyes distance (enL-enR)	34.31	2.86	34.16	2.91	0.14	(−0.04 to 0.33)
	Nose width (alL-alR)	33.64	2.70	33.50	2.72	0.72	(−0.10 to −0.25)
3	Nose prominence (prn-sn)	19.79	1.87	19.66	1.87	0.12	(0.00 to 0.24)
	Mid-face height (n-sn)	52.34	3.82	52.34	3.84	0.00	(−0.24 to 0.25)
	Mid-face height (sn-men)	48.30	3.49	48.11	3.41	0.18	(−0.03 to 0.41)
4	Maxilla angle (n-sn-pg)	162.45	5.69	162.32	5.63	0.13	(−0.23 to 0.50)
	Philtrum angle (prn-sn-Is)	127.12	8.78	127.35	8.85	0.22	(−0.80 to 0.35)
	Nose angle (n-prn-sn)	100.56	4.57	100.88	4.76	0.31	(−0.62 to −0.01)
5	Mandible angle(g-men-pg)	133.42	6.72	134.29	6.60	0.86	(−1.30 to −0.42)

SDB, sleep disordered breathing.

**Figure 2:**
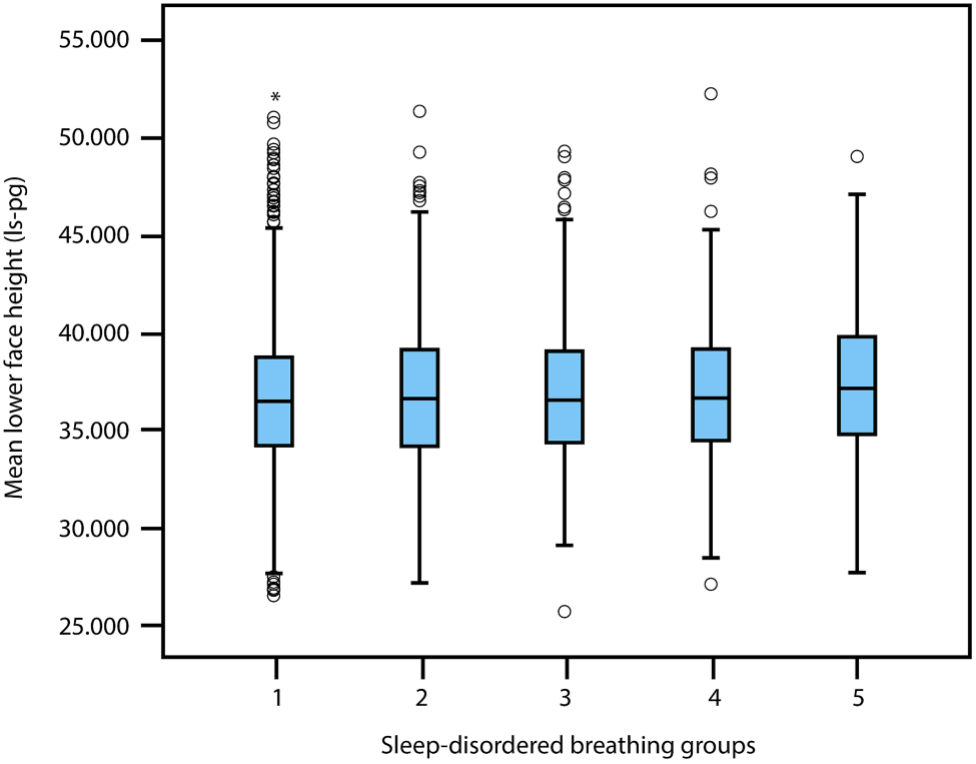
Mean±95% CI of lower face height (Is-pg) and 5 levels of sleep disordered breathing severity.

**Figure 3:**
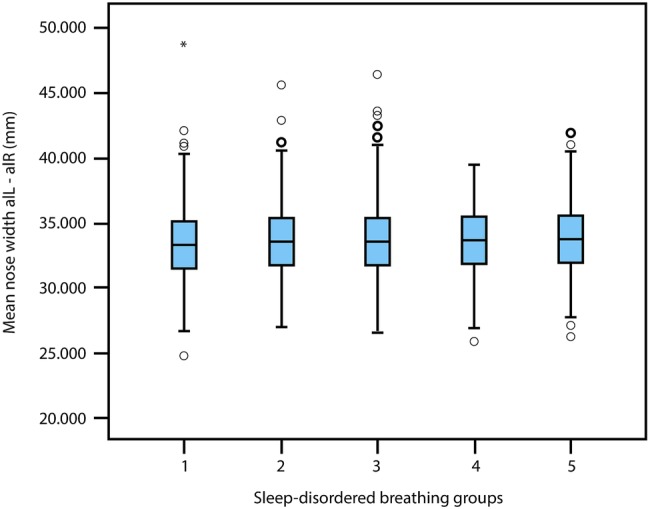
Mean±95% CI of nose width (alL-alR) and 5 levels of sleep disordered breathing severity.

**Figure 4:**
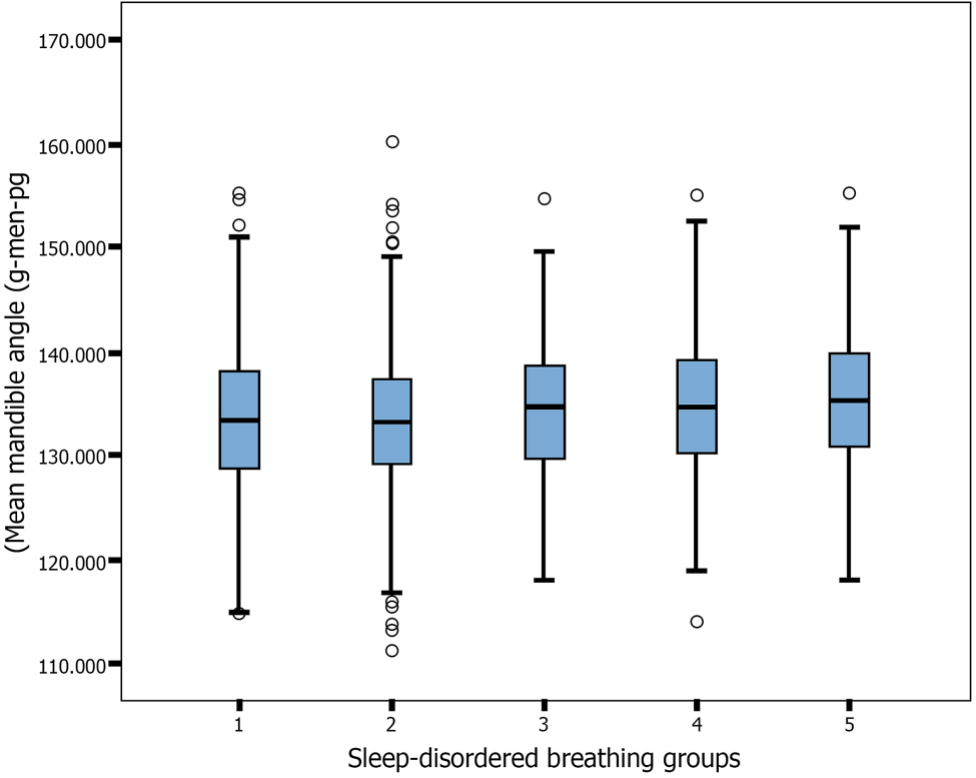
Mean±95% CI of mandible angle (g-men-pg) and 5 levels of sleep disordered breathing (SDB) severity. In each figure: 1=asymptomatic healthy; 2=children with early snoring, peak symptoms at 6 months; 3=children with early snoring, peak symptoms at 18 months; 4=children with late snoring and mouth breathing, but who remained asymptomatic until 4 years; 5=children with severe and sustained symptoms of SDB throughout childhood.

Systematic relationships between lower face height, nose width and mandible angle (mean±95% CI), with respect to the five levels of SDB severity, are illustrated in figures 2–4. Lower face height and mandible angle were consistently higher, and nose width was consistently lower, for those who experienced severe and sustained symptoms of SDB throughout childhood. ANOVA results for the lower face height, mandible angle and nose width are p=0.006, 0.000 and 0.004, respectively, with regard to the five levels of SDB groups.

### Five dimensions of face shape variables

Factor solutions collectively explaining 79% of the variance, with consistently strong factor loadings >0.5, were used to classify the 17 face shape variables into five dimensions (D_1_ to D_5_; table 4). D_1_ represented face height, which explained 32.6% of the variance. D_2_ represented the distance between the eyes with nose width, which explained 12.9% of the variance. D_3_ represented nose prominence with maxilla height (12.7% of the variance). D_4_ was the maxilla angle and nose with philtrum angle (11.2% of the variance), and D_5_ was the mandible angle (9.5% of the variance).

**Table 4:** Face shape dimensions extracted by factor analysis

	Percentage of variance explained	
	Per cent	Cumulative
Factor 1	32.6	32.6
Total face height (pg-men)		
Total face height (pg-g)		
Total face height (pg-n)		
Lower face height (Is-pg)		
Total face height (li-men)		
Mid-face height (Is-men)		
Mid-face height angle (exR-pg-exL)		
Factor 2	12.9	45.6
Outer eyes distance (exR-exL)		
Inner eyes distance (enL-enR)		
Nose width (alL-alR)		
Factor3	12.7	58.3
Nose prominence (prn-sn)		
Mid-face height (n-sn)		
Mid-face height (sn-men)		
Factor 4	11.2	69.5
Maxilla angle (n-sn-pg)		
Philtrum angle (prn-sn-Is)		
Nose angle (n-prn-sn)		
Factor 5	9.5	79
Mandible angle (g-men-pg)		

### Association of SDB and facial dimensions

Since SDB status was associated with BMI, logistic regression was used to examine the relationships between the facial dimensions and SDB status adjusted for BMI. A binary logistic regression model was constructed (the results are presented in table 5), using the principal component scores for the five dimensions extracted by factor analysis as the predictor variables. Four dimensions were significantly associated with SDB. The odds of the children exhibiting symptoms of SBD increased significantly with respect to D_5_—mandible angle (OR 1.11, 95% CI 1.04 to 1.19), and D_1_—face height (OR 1.09, 95% CI 1.02 to 1.16). In contrast, an increase in D_2_—distance between the eyes with nose width (OR 0.90, 95% CI 0.84 to 0.97), and an increase in D_3_—nose prominence with mid-face height (OR 0.93, 95% CI 0.86 to 0.99), was associated with reduced odds of SDB. The dimensions D_4_—maxilla angle, nose with philtrum angle, was not significantly associated with SDB. An increase in the BMI was associated with increased odds of SDB (OR 1.03, 95% CI 1.01 to 1.05).

**Table 5:** Binary logistic regression model using five face shape dimensions

Predictor	OR	p Value	95% CI	
D_1_ face height	1.09	0.011	1.02	1.16
D_2_ eyes distance with nose width	0.90	0.005	0.84	0.97
D_3_ nose prominence with mid-face height	0.93	0.028	0.86	0.99
D_4_ maxilla angle, nose with philtrum angle	1.05	0.162	0.98	1.12
D_5_ mandible angle	1.11	0.001	1.04	1.19
BMI	1.03	0.003	1.01	1.05

BMI, body mass index.

### Superimposition of average faces

**Figure 5:**
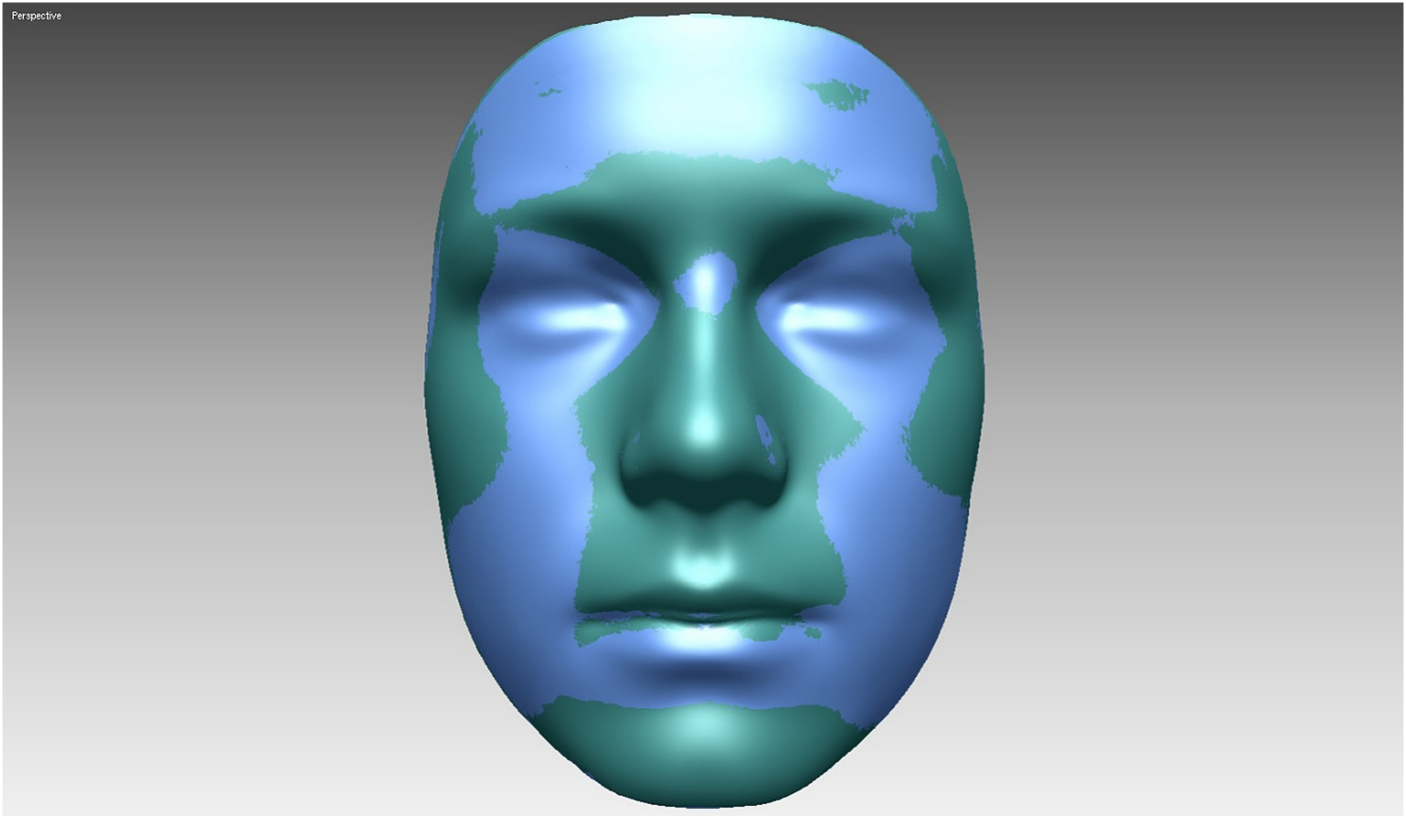
Superimposition of average facial shells of sleep disordered breathing and healthy children.

**Figure 6:**
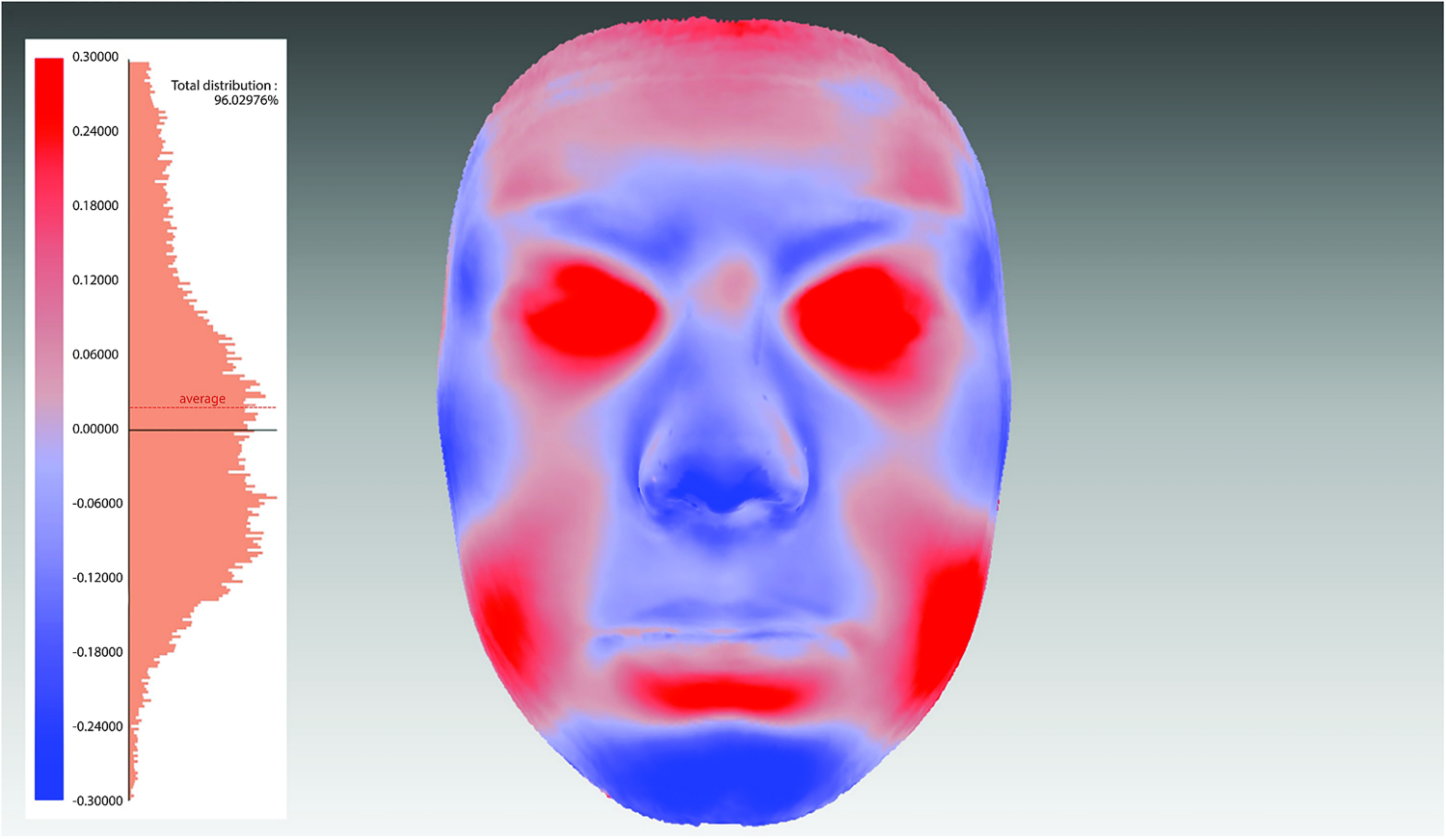
Colour maps and histogram plots to assess facial differences between children with and without sleep disordered breathing (SDB). The pink areas represent no difference in the SDB and non-SDB children (0 mm). The blue areas represent less prominent chin and nose in SDB children (0.1–0.6 mm), while the deeper blue represents greater facial retrusion in the SDB groups. The red areas are those prominent features in the SDB face.

Superimposed surface-based average faces of SDB and healthy children are presented in figure 5, while the colour maps in figure 6 show morphological differences between the groups. As the figures illustrate, healthy children tended to have slightly bigger noses, more prominent mandibles, cheeks and foreheads when compared to SDB children.

## Discussion

Previous analyses of the variability in facial anomalies associated with the development of SDB among children (aged 0–18 years) provide inconsistent and conflicting results. Consequently, the concept that nasal obstruction and associated mouth breathing influence craniofacial development and morphology, and are related to SDB in children, is controversial.^24^ Misleading conclusions may have been drawn in previous studies because the sample sizes were too small, providing insufficient statistical power; and the cases with SDB and the asymptomatic controls were not necessarily equivalent with respect to their demographic and other attributes (eg, equal proportions of cases and controls by gender, age, obesity and clinical history). If the study groups were not demographically equivalent, then the differences between the face shape variables could potentially be confounded by factors other than SDB. In accordance with the design of an effective study,^45^ we used a large sample size (1693 males and 1893 females) all of whom were the same age (15 years) in order to provide sufficient statistical power; interpreted effect sizes in addition to p values; ensured that the SDB and healthy children were demographically equivalent; and controlled for confounding variables (BMI and gender). Furthermore, none of the children in this study had their tonsils and/or adenoids removed, while the possible confounding effect of obesity was ascertained.

The possible effects of obesity may be a confounding factor leading to conflicting observations in previous studies. However, our findings support Verhulst *et al*,^21^ who concluded that obese children are at a higher risk of developing SDB.

Using factor analysis, we established that 17 variables measured by use of a three-dimensional facial scan could be reduced to five dimensions of face shape. We established consistent outcomes using binary logistic regression to conclude that, among children with SDB relative to healthy children, the mandible was retrognathic, the face height dimensions were significantly higher and the nose prominence and nose width dimensions were consistently lower. The mandible among the SDB children was found to be significantly less prominent and in a posterior position relative to the maxilla, supporting previous evidence that the prevalence of retrognathic mandible in mouth breathing children is higher than in nasal breathing children.^46^ Increased total and lower face height has previously been reported among children with SDB.^18^ ^47–49^ Nasal obstruction associated with mouth breathing is assumed to lead to a downward and backward rotation of the mandible, and to an increase in anterior face height.^24–26^ ^29^ This is consistent with our findings that face height measurements were higher in SDB children when compared to healthy asymptomatic children. We also found that the nose prominence dimension was lower in children with SDB relative to asymptomatic children. This is consistent with the findings of Zettergren-Wijk *et al*,^48^ who reported that the nose was less pronounced in a small sample of children (10 boys and 7 girls) with OSA when compared with controls. It is suggested that nose prominence could reflect a comparatively short anterior cranial base. In contrast, we found no statistical evidence to determine a significant difference between SDB and healthy asymptomatic children with respect to maxillary prognathism, consistent with the findings of Zettergren-Wijk *et al*.^48^ Overall correlations between SDB severity and facial morphology were indicated in this study, which supports the findings of Wenzel *et al*,^50^ who reported a more retrognathic mandible in association with increasing severity of breathing disorders.

The limitation of this study is that SDB was assessed through parental reports of SDB's hallmark symptoms (snoring, apnoea and mouth breathing). Although PSG is considered the ‘gold standard’ for assessing SDB, the time, expense, possible selection bias of those undergoing PSG and possible methodological changes over time rendered it unfeasible for epidemiological purposes in a large longitudinal cohort study; on the other hand, the five patterns of symptoms of SDB defined in this study were assumed to be reliable, because they are correlated with the outcomes of PSG examination.^36^ ^51^

## Conclusion

Consistent evidence was provided using binary logistic regression and three-dimensional average face superimposition to confirm the hypothesis that SDB (snoring, apnoea and mouth breathing) among a cohort of 15-year-old children was associated with (1) an increase in face height; (2) a decrease in nose prominence; (3) a decrease in nose width; and (4) a retrognathic mandible. There was, however, no statistical evidence to determine if the prevalence and severity of SDB was associated with an increase or decrease in the angle of the maxilla. However, evidence was found to indicate an association between increased BMI and the prevalence of SDB symptoms.

Since SDB has serious consequences for long-term health and quality of life, early diagnosis of SDB is essential. Healthcare professionals can play an important role in the early diagnosis of SDB, recognising distinct facial morphologies of long face, reduced nose prominence and a retrognathic mandible and referring these children to specialists for further assessment of SDB clinical symptoms.
